# Advancing crisis readiness through progressive simulation in undergraduate medical education

**DOI:** 10.1186/s41077-026-00427-w

**Published:** 2026-02-26

**Authors:** Rebekah Cole

**Affiliations:** Department of Health Professions Education, Uniformed Services University, 4301 Jones Bridge Rd, Bethesda, MD 20814 USA

**Keywords:** Progressive simulation, Disaster response, Medical education, Experiential learning

## Abstract

As healthcare professionals worldwide face increasing demands from disasters, mass casualty incidents, and other complex emergencies, medical education must prepare students to perform in high-pressure, resource-limited environments. Traditional simulation experiences are often episodic and narrowly focused, limiting their ability to cultivate the longitudinal development of clinical skills, leadership, and adaptability. This innovation report presents a four-year, progressive simulation curriculum designed to build crisis readiness across the undergraduate medical education continuum. Informed by experiential learning theory and cognitive load theory, this innovative model scaffolds learners through staged simulation experiences that grow in complexity and responsibility. From early roleplay with peers to immersive field exercises and capstone leadership scenarios, the simulation curriculum integrates clinical, communication, and team-based competencies in increasingly dynamic environments. This longitudinal approach aligns with principles of competency-based medical education and offers a scalable, transferable model for enhancing readiness and resilience in future physicians.

## Introduction

Simulation-based education is an essential component of medical training, in which learners have the opportunity to acquire and refine their clinical, communication, and decision-making skills in a safe and controlled environment [1]. However, traditional simulation formats are often episodic and narrowly focus on developing a specific skill set. These constraints are arguably a limitation that reduces simulation-based education’s potential to prepare students for challenges such as disaster response, prolonged field care, or dynamic prehospital environments, where uncertainty, resource scarcity, and leadership demands are high. With the frequency and impact of natural disasters, pandemics, mass casualty incidents, and other complex humanitarian emergencies increasing worldwide, there is a growing imperative to design simulation-based curricula that build medical students’ adaptive capacity, interprofessional coordination, and decision-making under pressure [2, 3].

Current-day simulation-based education calls for a more holistic and longitudinal use of simulation that mirrors real clinical care [1]. One such modality is progressive simulation, an instructional framework that scaffolds simulation experiences across increasing levels of complexity and responsibility over time. Grounded in experiential learning theory and cognitive load theory, progressive simulation emphasizes cumulative learning, adaptive expertise, and situated decision-making [4, 5].

While Graduate Medical Education programs have integrated elements of progressive simulation through mastery learning and milestone-based assessments, no undergraduate medical curricula have adopted a structured, four-year model of progressive simulation. This innovation report introduces a longitudinal simulation curriculum developed at a military medical school and examines its applicability to both military and civilian training contexts.

### Positioning this innovation

Current simulation-based education initiatives often include bootcamps, summative Objective Structured Clinical Examinations (OSCEs), or scenarios that are effective for specific skills but lack continuity and developmental sequencing [6, 7]. Programs such as TeamSTEPPS training, disaster preparedness modules, and communication-focused standardized patient encounters contribute valuable components, but do not incrementally develop learner competencies over time [8–10]. This innovation expands on these approaches by embedding progressive simulation throughout all four years of medical school, with increasing demands on clinical reasoning, teamwork, and leadership. Ultimately, this curriculum design enables integration into competency-based medical education (CBME) frameworks by providing longitudinal data on learner performance, resilience, and readiness [11].

### Problem addressed

A critical gap in medical education is its limited capacity to prepare students for dynamic, high-pressure prehospital environments that demand rapid decision-making and adaptability. Traditional didactic learning and short-duration simulations do not adequately develop the cognitive, procedural, and leadership skills required for these settings [12, 13], Although simulation-based education has demonstrated effectiveness in developing clinical competencies [14], few undergraduate medical curricula have implemented a deliberately structured, longitudinal progressive simulation program that spans all four years of training; most reported interventions remain short-term, course-based, or focused on a single domain rather than a fully integrated program-wide model [15, 16].

### Approach

This progressive simulation model integrates four full-scale immersive simulations that are built upon one another to enhance clinical readiness, leadership, and resilience in low-resource, high-stress environments. We adapted scenarios from existing military operational training templates [17] and structured around best practices of simulation design, including clearly defined learning objectives, pre-briefing, fidelity selection, learner role assignments, scenario flow, and structured debriefing [18–22]. In addition, the curricula were informed by military training protocols and disaster medicine and trauma simulation models to enhance realism and scalability. This structured approach, guided by experiential and cognitive load theory [23], ensured that the curriculum was built on recognized educational standards. The progressive simulation curriculum undergoes regular evaluation to determine its impact on students’ learning and performance.

Each iteration of the progressive simulation involves approximately 200 learners, who complete the exercise simultaneously but in smaller groups of 20–25 students.This number includes approximately 180 U.S. medical students who engage in each simulation, 20–25 U.S. nursing students (during third and fourth year simulations), and 15–20 international military medical trainees (during the fourth year simulation). The international participants are typically medical trainees from other military medical institutions, participating as part of an interprofessional and intercultural exchange program. Faculty are recruited through existing university faculty networks, partnerships with military treatment facilities, federal disaster response agencies, and academic institutions and research centers with expertise in emergency and disaster medicine. Faculty-to-learner ratios are intentionally structured to maintain close supervision and coaching across all simulation phases. The second-year simulation operates at approximately a 1:3 ratio (supported by ~ 30 faculty across four iterations serving 180 medical students), the 48-hour third-year simulation maintains a 1:4 ratio (~ 35 faculty across four iterations serving 200 medical and nursing students), and the fourth-year capstone field exercise maintains a 1:5 ratio (~ 75 faculty across two iterations serving 220 medical, nursing, and international students). Collectively, these ratios ensure consistent access to expert guidance even as the scale and complexity of training increase.

#### First-year: foundational patient experience

During their first year of medical school, 175 first year medical students portray patients in a simulated wartime setting, while fourth-year students serve as healthcare professionals. They are guided by 6–8 faculty coaches, who prepare them via a structured curriculum to accurately portray various illnesses and injuries in the simulation (as described in the subsequent section on fourth-year simulation). This immersive role reversal fosters early professional identity formation and introduces students to the complexities of high-stress, resource-limited prehospital care. By adopting the patient perspective, first-year students report developing empathy, experiencing vulnerability and dependency, and gaining insight into patient–provider dynamics, which are an essential foundation for patient-centered communication. Casting peers as patients allows for realistic, operationally relevant scenarios (such as wartime trauma) in a cost-effective and easily replicable manner. Recent research shows that role playing the patient role may increase medical knowledge, deepens empathy, strengthens self-awareness and professional identity, and enhances understanding of power dynamics, making peer-based patient roles a powerful tool for cultivating empathy and leadership [24–26]. During the first-year simulation, students are not formally assessed; rather, this stage is designed to foster empathy, insight into the patient experience, and professional identity formation.

#### Second-year: introductory clinical simulation

Students participate in a one-day trauma simulation involving five 30-minute scenarios. Teams perform emergency procedures on manikins and task trainers in a simulated prehospital outdoor environment while receiving real-time feedback from experienced physicians. This stage focuses on basic clinical skills, and team communication under time constraints. It introduces students to high-stress decision-making and teamwork in prehospital care. Beginning in Year 2 and continuing through Years 3 and 4, students are assessed using standardized Bushmaster Assessment Cards, which are behaviorally anchored tools that have been systematically developed and validated [27]. These cards combine domain-specific checklists with rating scales to evaluate core competencies including clinical decision-making, leadership, teamwork, communication, and professionalism.

In the second, third, and fourth year simulations, faculty raters complete the assessment cards in real time and provide immediate formative feedback through structured debriefing.

#### Third-year: prolonged patient care simulation

Expanding on the skills learned during their second year, this 48-hour prehospital prolonged care simulation challenges students to manage patients in small teams of 8–10 students with limited resources and sleep. This simulation introduces collaboration with nursing students, which simulates interprofessional teamwork. The third-year medical students are assessed on clinical skills, leadership, teamwork, and communication. This simulation fosters endurance and resilience while simulating real-world, low-resource constrained environments.

#### Fourth-year: capstone medical leadership scenario

The culminating five-day simulation requires fourth-year medical students to lead 20–25-person interprofessional teams composed of medical, nursing, and international trainees who care for live simulated patients, who are played by first year medical students. These teams must manage multiple patients, including two mass-casualty scenarios, in a high-stress prehospital environment with significant logistical constraints. Faculty are charged with ensuring the psychological safety of the students during these stressful iterations. Throughout the simulation, students coordinate team roles, allocate scarce resources, perform mass-casualty triage, and make real-time leadership decisions. During the fourth-year capstone simulation, the same assessment tool is used for summative evaluation, supporting entrustment and progression decisions. Faculty raters, trained in the use of the tool, are embedded with student teams to ensure consistent evaluation and feedback across multiple scenarios. This standardized assessment approach allows for reliable longitudinal data collection in complex, high-stakes environments.

#### Facilitation and logistics

Implementing this model of longitudinal, progressive simulation involves coordination of curricular design, faculty and student travel and scheduling logistics, and safety protocols. Each simulation is embedded within the medical school calendar to ensure protected time for participation, with planning beginning 10–12 months in advance. Scenarios are scripted by an interdepartmental faculty team who are experts in emergency medicine, family medicine, surgery, nursing, and military operational medicine. During each simulation, faculty assignments are determined by learner ratios and event length, with multi-shift coverage required to sustain continuous scenarios.

In the 48-hour third-year exercise, students are organized into interprofessional teams that balance clinical duties with scheduled on-site sleep cycles, which students manage within their teams. Casualty encounters are introduced at irregular intervals to simulate unpredictability and uncertainty, while faculty rotate through staggered shifts to provide supervision and feedback without breaking immersion. Logistical staff manage simulated supply constraints to replicate austere field conditions. Although the 48-hour third-year simulation is designed as a single, continuous operational scenario, it is segmented into multiple operational phases to support structured reflection and formative feedback. Faculty facilitate After Action Reports (AARs) approximately every 4–6 h, allowing teams to reflect on performance, identify strengths and gaps, and adjust their approach while maintaining scenario immersion [28].

The five-day capstone exercise for fourth-year students represents a large-scale field deployment that integrates medical students, nursing students, graduate behavioral health students, and international medical trainees. Students sleep on-site, with logistical staff managing meals and equipment to sustain continuous operations. Patient flow spans day and night cycles, with planned patient surges designed to test students’ leadership and stress management capacity. Faculty facilitators, simulated patients, and moulage teams rotate in 8–12 h shifts. Student-led AARs follow each scenario to foster reflection and recalibration. During this capstone simulation, students receive a summative assessment reflecting their performance over all four years of simulation training.

This progressive, multi-day simulation framework replicates real-world prehospital environments and fosters medical students’ clinical skills, communication, and leadership under stress. Across all four years, formative and summative assessments are conducted through standardized checklists, direct observation, and structured AARs. This longitudinal assessment of students’ professionalism and skills enables tracking of student growth and development, serving as a foundation of CBME.

#### Faculty training

Facilitating progressive simulation requires specialized faculty training in simulation pedagogy and feedback delivery within immersive simulation. Structured faculty development workshops and ongoing mentorship have improved faculty confidence and enhanced the student learning experience.

#### Assessment standardization

Capturing longitudinal growth in non-technical competencies has been challenging. Future iterations will incorporate artificial intelligence (AI)-driven analytics to assess clinical reasoning, stress responses, leadership development, and communication patterns, including automated coding of radio and verbal communications, dynamic analysis of patient triage decisions, leadership role tracking, and stress biomarker integration. These tools may result in richer feedback and longitudinal assessment capabilities.

#### Generalizability and broader applications

This progressive simulation model has broad applicability to training in emergency medicine, rural care, and disaster preparedness. This progressive simulation model also aligns with CBME by enabling entrustment decisions and holistic assessment of learner progression, as it provides repeated opportunities for learners to demonstrate integrated competencies such as clinical reasoning, leadership, teamwork, and communication under realistic, high-stress conditions. Because these simulations are situated longitudinally, faculty can directly observe performance trends over time, supporting more defensible entrustment decisions. This approach is consistent with CBME, where decisions about readiness are made after reviewing multiple data points collected in authentic clinical contexts rather than solely by classroom-based checklist assessments. Partnerships with community organizations and global health initiatives can extend this model beyond academic settings.

### Next Steps

Future curriculum development will focus on integrating stress inoculation techniques (such as mindfulness training and resilience coaching) [29, 30] and leveraging AI-driven formative feedback to tailor individualized learning. Additionally, scaling this program to multiple institutions will enable further evaluation of its impact and best practices for broader adoption in medical education.

## Conclusion

This four-year progressive simulation model represents an evidence-informed approach to developing crisis-ready physicians. It addresses current gaps in simulation pedagogy and prepares medical students for real-world challenges. Critically, this model also integrates sustained interprofessional education, bringing together medical, nursing, and international learners in high-stress, resource-limited scenarios that mimic authentic environments. Embedding this collaborative training throughout undergraduate medical education may strengthen learners’ readiness to lead and optimally perform in future crisis settings.

## Data Availability

The datasets used and analyzed during the current study are available from the corresponding author on reasonable request.
